# Intrinsic hypoexcitability of thalamic nucleus reuniens inputs to hippocampal CA1 in the 5xFAD mouse model of amyloidosis

**DOI:** 10.1002/alz70855_106177

**Published:** 2025-12-24

**Authors:** Chengju Tian, Isabel Reyes, Mohankumar Thangavel, Arjun V. Masurkar

**Affiliations:** ^1^ New York University Grossman School of Medicine, New York, NY, USA

## Abstract

**Background:**

The nucleus reuniens of thalamus (nRT) plays important roles in memory and provides direct excitatory input to the distal dendrites of CA1 pyramidal neurons (PNs). While it is well known that the entorhinal cortex (EC) excitatory inputs to CA1 PN distal dendrites are vulnerable to dysfunction, it is unclear how nRT→CA1 inputs are compromised, and whether any dysfunction is intrinsic to nRT or at the nRT→CA1 synapse.

**Method:**

Recordings were performed in 5‐11 month old 5xFAD and WT female mice using in vitro whole cell patch clamp. To measure intrinsic excitability of nRT, acute whole brain coronal slices were prepared and electrodes were targeted to nRT principal neurons. Subthreshold and suprathreshold properties were evaluated in current clamp. For examination of nRT→CA1 synapses, nRT of female 5xFAD and WT mice (5‐11 month old) were injected with an AAV expressing channelrhodopsin ChrR2. After two weeks, acute transverse hippocampal slices were prepared and whole cell patch clamp recordings were performed on CA1 PNs. Synaptic responses were measured in current clamp mode, with nRT axons optogenetically activated by blue LED light. Excitatory postsynaptic potentials (EPSPs) were measured with inhibition blocked.

**Result:**

All data is n = 9‐12/group. The nRT neurons showed two modes of firing: regular tonic firing or a low‐threshold burst potential with superimposed spikes. Compared to WT, nRT neurons in 5xFAD mice showed reduced rates of tonic regular spiking (23.6 vs. 42.7Hz, *p* = 0.04), a shorter low‐threshold burst potential half‐width (98 vs. 135ms, *p* = 0.029), and reduced spikes per burst (4.7 vs. 5.3, *p* = 0.049). In WT, optical activation of nRT axons produced small EPSPs in CA1 PNs below 1mV that were unchanged in amplitude across the transverse axis of CA1. Moreover, there was no statistically significant change in their amplitude when compared to 5xFAD mice.

**Conclusion:**

Excitatory flow from nRT to CA1 is reduced in the setting of amyloidosis due to reduced intrinsic low threshold burst and spike firing within the nRT. In contrast, the relative stability of nRT→CA1 synapse itself suggests that this pathway may serve as a salvage mechanism to excite distal CA1 dendrites in the setting of compromised EC→CA1 excitation.

